# Polyindole-Functionalized RGO-NiFe_2_O_4_-SiO_2_ Nanocomposite: A Dual-Functional Nanomaterial for Efficient Antimony Adsorption and Subsequent Application in Supercapacitor

**DOI:** 10.3390/polym16213084

**Published:** 2024-10-31

**Authors:** Mohd Shoeb, Fouzia Mashkoor, Mohmmad Naved Khan, Changyoon Jeong

**Affiliations:** School of Mechanical Engineering, Yeungnam University, Gyeongsan 38541, Republic of Korea; mshoeb@yu.ac.kr (M.S.); fmashkoor@yu.ac.kr (F.M.); navedkhan@yu.ac.kr (M.N.K.)

**Keywords:** adsorption, reduced graphene oxide, NiFe_2_O_3_-SiO_2_, polyindole, antimony, supercapacitor

## Abstract

Effective wastewater treatment remains a critical challenge, especially when dealing with hazardous pollutants like antimony (Sb(III)). This study addresses this issue by using innovative nanocomposites to remove Sb(III) ions from water, while simultaneously repurposing the spent adsorbents for energy storage applications. We developed reduced graphene oxide-NiFe_2_O_3_-SiO_2_-polyindole nanocomposites (RGO-NiFe_2_O_3_-SiO_2_-PIn NCs) via a hydrothermal synthesis method, achieving a high removal efficiency of 91.84% for Sb(III) ions at an initial concentration of 50 mg/L at pH 8. After adsorption, the exhausted adsorbent was repurposed for energy storage, effectively minimizing secondary pollution. The Sb(III)-loaded adsorbent (RGO-NiFe_2_O_3_-SiO_2_-PIn@SbO_x_) exhibited excellent performance as an energy storage material, with a specific capacitance (C_s_) of 701.36 F/g at a current density of 2 A/g and a retention rate of 80.15% after 10,000 cycles. This dual-purpose approach not only advances wastewater treatment technologies but also contributes to sustainable and economical recycling practices, particularly in the field of energy storage.

## 1. Introduction

Water is one of the most valuable natural resources in the 21st century, essential for supporting human civilization [1]. However, the rapid growth of the global population, alongside agricultural, industrial, and urban expansion, has created a significant imbalance between water availability and demand. Industrial processes such as metal manufacturing, mining, and power generation contribute to the contamination of water with various pollutants, including toxic heavy metals [2,3]. Among these, antimony (Sb) is a key concern due to its widespread use in industries like battery production and flame retardants, leading to increased discharge into the environment. Antimony compounds, particularly in their trivalent form, are highly toxic and comparable to arsenic in terms of health risks. This heavy metal is linked to severe health problems, including cancer, and is recognized as a top-priority contaminant by both the EU and EPA. Therefore, efficient removal of antimony from wastewater is critical for environmental protection and human health.

A variety of treatments are employed to eliminate toxic substances from contaminated water, including electrodialysis, chemical precipitation, photocatalysis, and membrane filtration [4,5]. However, these traditional approaches often face significant limitations, such as high energy consumption, incomplete treatment, the need for expensive equipment, and the production of sludge or solid waste that requires further disposal. Additionally, they may not be efficient for treating large volumes of wastewater, limiting their scalability. In contrast, adsorption has gained recognition as a highly effective and environmentally friendly method for removing heavy metals, including antimony, from water [6,7]. This process is not only cost-effective but also simple in design, flexible in operation, and capable of recovering adsorbents [8,9]. The success of adsorption, however, hinges on the quality of the material used as an adsorbent, which plays an important role in overall adsorption efficiency [10]. Recent studies have focused on developing advanced adsorbents with enhanced adsorption capacities, better regeneration potential, and faster separation from large volumes of water aiming for more efficient and scalable water treatment solutions. Innovations such as hybrid adsorbents, which combine the benefits of multiple materials and improve separation efficiency, and functionalized nanomaterials are being developed to improve water treatment processes. Continuous advancements in adsorbent materials are essential to address the growing demand for more scalable and efficient solutions for water purification.

Various nanocomposites have been widely employed to remove contaminants from water systems, including carbon, polymers, metal oxides, and inorganic–organic materials. Among these, conducting polymers, such as polyindole (Pln), have garnered interest due to their facile production, economic viability, and adjustable redox characteristics. Pln, composed of indole units with fused benzene and pyrrole rings, is recognized for its high thermal stability, chemical versatility, and ability to chelate metal ions [11]. However, limitations in its mechanical properties and stability have hindered its broader application in environmental remediation [12]. To enhance polyindole’s properties, researchers have incorporated it into nanocomposites with materials like metal oxides. The integration of polyindole with metal oxides such as NiFe_2_O_3_-SiO_2_ offers several key advantages. NiFe_2_O_3_ (nickel ferrite) is a magnetic metal oxide known for its excellent stability, reusability, and effective adsorption capabilities, particularly for heavy metals. Its magnetic properties facilitate easy separation from water using an external magnetic field, making it ideal for wastewater treatment applications [13]. SiO_2_ (silica) contributes significantly to the composite’s performance by providing structural support and increasing the surface area available for adsorption [14]. Silica’s high porosity and chemical stability allow for the efficient immobilization of polyindole and NiFe_2_O_3_, enhancing the mechanical strength and durability of the resulting nanocomposite under harsh environmental conditions. The incorporation of SiO_2_ not only improves the physical integrity of the composite but also promotes better dispersion of the metal oxide particles within the polymer matrix, maximizing their contact with contaminants.

This combination enhances the nanocomposite’s adsorption capacity by creating more active sites for binding contaminants like antimony. After modifying polyindole with NiFe_2_O_3_-SiO_2_, anchoring the composite onto reduced graphene oxide (RGO) further improves its functionality. RGO, characterized by its two-dimensional architecture and large surface area, provides additional sites for the adsorption of contaminants, complementing the enhanced capabilities of the modified polyindole. The presence of oxygen-comprising functional groups on RGO enhances interactions with metal ions, thereby increasing adsorption capacity and selectivity [15]. The combination of polyindole, NiFe_2_O_3_-SiO_2_, and RGO results in a synergistic effect that boosts the overall stability, reactivity, and adsorption efficiency of the nanocomposite. This integrated material shows promise for efficient and scalable solutions in wastewater purification, particularly for the removal of toxic heavy metals like antimony.

Addressing the environmental risks associated with improper disposal of used adsorbents, such as RGO-NiFe_2_O_3_-SiO_2_-PIn, is crucial for protecting ecosystems. Effective management of these spent materials is essential to prevent their harmful effects and buildup in an environment. The development of innovative strategies to capture and reuse contaminants from effluent not only improves sustainability but also generates valuable resources. A compelling solution entails the conversion of exhausted adsorbents comprising antimony into highly efficient electrode materials for supercapacitors [16,17,18]. Antimony is highly regarded for its electrochemical properties, which include high capacity, conductivity, thermal stability, optimal voltage, and rapid ion diffusion [19]. This unique combination of traits has sparked considerable interest in using antimony compounds for energy storage applications. Recent research has revealed impressive results. For instance, studies on antimony oxide graphene nanoparticles achieved a remarkable specific capacitance (C_s_) of 98 F/g at a current density of 0.1 A/g, demonstrating excellent rate performance and durability [19]. Similarly, antimony–tin oxide nanomaterials have shown a C_s_ of 84.53 F/g in supercapacitor applications [20]. Additionally, devices utilizing antimony–titanium carbide MXene composites have exhibited exceptional capabilities, boasting a C_s_ of 184.72 F/g at 1 A/g and outstanding cycling stability [21]. Recognizing the importance of Sb(III) and RGO-NiFe_2_O_3_-SiO_2_-PIn in energy storage, this research aims to align with the “waste-to-wealth” concept. By repurposing waste materials into valuable energy solutions, this approach not only addresses the challenges of wastewater treatment but also contributes to the advancement of sustainable energy technologies.

In this work, hydrothermally synthesized RGO-NiFe_2_O_3_-SiO_2_-PIn was employed for the removal of antimony from wastewater. Interestingly, the expended adsorbent (RGO-NiFe_2_O_3_-SiO_2_-PIn@SbOx) was subsequently investigated for its potential in energy storage as an electrochemical supercapacitor to avoid secondary pollution caused by the spent adsorbent. The RGO-NiFe_2_O_3_-SiO_2_-PIn shows a removal efficiency of 91.84% at optimized pH and adsorbent dose and an equilibrium time of 90 min. The electrochemical supercapacitor analysis designed from the Sb-adsorbed RGO-NiFe_2_O_3_-SiO_2_-PIn (RGO-NiFe_2_O_3_-SiO_2_-PIn@SbOx) presented a 701.36 F/g at a current density of 2 A/g with a retention rate of 80.15% after 10,000 cycles. The primary objective of this study is to transform detrimental waste discharged into water bodies into a value-added product. Through this approach, the adsorption technique is effectively employed, contributing to a decline in water contamination while generating a valuable end product. With this objective in mind, the integration of the adsorption technique and supercapacitor has been carried out to establish a more sustainable and environmentally friendly approach for both wastewater treatment and energy storage.

## 2. Experimental

### Synthesis of RGO-NiFe_2_O_3_-SiO_2_-PIn Nanocomposite

**Figure 1:**
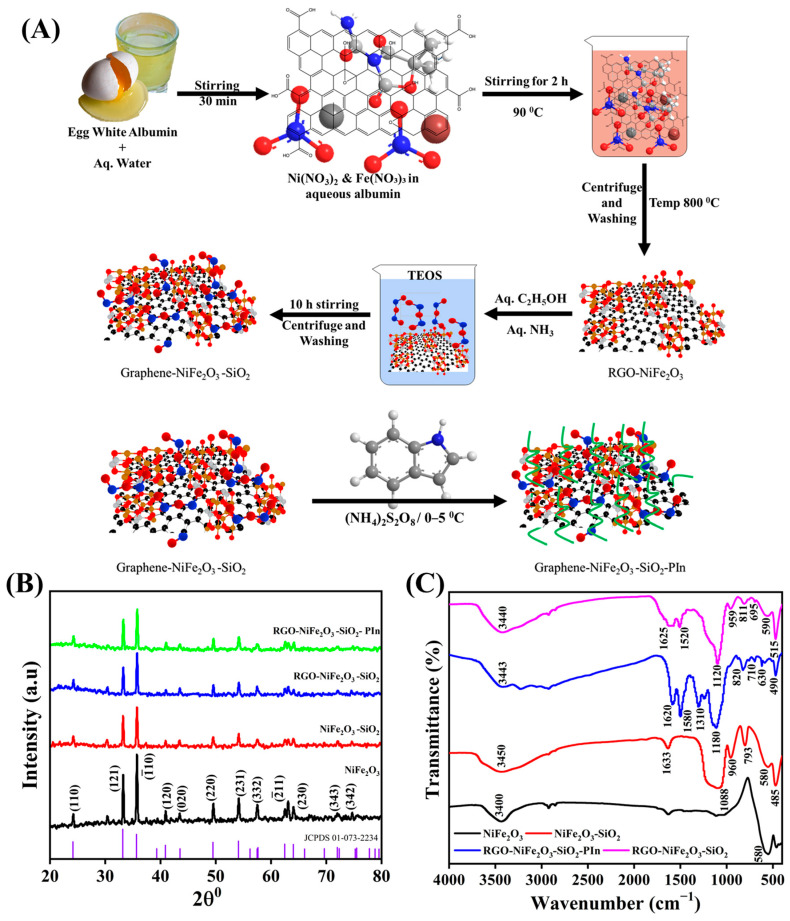
(**A**) Synthesis scheme of RGO-NiFe_2_O_3_-SiO_2_-PIn NCs: (**B**) XRD and (**C**) FTIR of NiFe_2_O_3_, NiFe_2_O3-SiO_2_, RGO-NiFe_2_O_3_-SiO_2_, and RGO-NiFe_2_O_3_-SiO_2_-PIn.

The synthesis of RGO-NiFe_2_O_3_-SiO_2_-PIn begins with preparing RGO-NiFe_2_O_3_ using a bio-template approach [22]. Egg white albumin is used as a natural capping and stabilizing agent. In the first step, egg white albumin is dissolved in deionized water and stirred for 30 min to form a homogeneous mixture. Afterward, nickel nitrate (Ni(NO_3_)_2_).6H_2_O (50 mL; 0.05 M) and iron nitrate (Fe(NO_3_)_3_).9H_2_O (50 mL; 0.1 M) are introduced into the aqueous albumin solution. This results in forming a precursor mixture containing nickel and iron ions. The solution is stirred for 2 h at 90 °C to facilitate the interaction of the metal ions with the albumin and graphene oxide, allowing the formation of metal–graphene complexes. Subsequently, the mixture is centrifuged to remove impurities and then washed. The resulting material is calcinated at 800 °C to reduce the graphene oxide and form nickel ferrite (NiFe_2_O_3_) nanoparticles. This process yields the composite RGO-NiFe_2_O_3_, where nickel ferrite is uniformly distributed across the reduced graphene oxide surface. In the second step, SiO_2_ is integrated into the RGO-NiFe_2_O_3_ structure using the sol–gel method. Tetraethyl orthosilicate (TEOS; 5 mL) is dissolved in ethanol (C_2_H_5_OH), followed by the addition of aqueous ammonia (NH_3_ ~ pH-8) to initiate hydrolysis and condensation reactions. The RGO-NiFe_2_O_3_ material is then added to this TEOS-containing solution, and the entire mixture is stirred continuously for 10 h, promoting the deposition of SiO_2_ onto the RGO-NiFe_2_O_3_ surface. After the completion of the reaction, the product is separated via centrifugation and thoroughly washed with water and ethanol to remove any residual reactants. This yields the composite RGO-NiFe_2_O_3_-SiO_2_, where silica nanoparticles are integrated with the nickel ferrite–graphene structure. In the final step, PIn is introduced onto the RGO-NiFe_2_O_3_-SiO_2_ nanocomposite through oxidative polymerization. A solution of ammonium persulfate ((NH_4_)_2_S_2_O_8_), an oxidizing agent, is prepared and cooled to 0–5 °C. The RGO-NiFe_2_O_3_-SiO_2_ composite is then added to this solution. The low temperature ensures controlled polymerization of indole monomers, allowing them to form polyindole chains on the surface of the composite. The presence of ammonium persulfate initiates the polymerization by generating free radicals, which react with indole monomers, forming polyindole (PIn). After the polymerization is complete, the product is again centrifuged and washed to remove unreacted species and by-products, resulting in the final product, RGO-NiFe_2_O_3_-SiO_2_-PIn (Figure 1A). The detailed experimental procedure of the adsorption of Sb(III) and the energy storage application is given in the Supplementary File.

## 3. Results and Discussion

Figure 1B presents the X-ray diffraction (XRD) patterns for NiFe_2_O_3_, NiFe_2_O_3_-SiO_2_, RGO-NiFe_2_O_3_-SiO_2_, and RGO-NiFe_2_O_3_-SiO_2_-PIn nanocomposites. The diffraction peaks, observed at 2θ values of 24.18°, 33.19°, 35.67°, 39.31°, 40.89°, 43.54°, 49.49°, 54.11°, 56.19°, 57.64°, 62.47°, 64.05°, 72.34°, 75.51°, and 77.79°, correspond to the reflection (110), (121), $\bar{(1}10)$ (120), (020), (220), (231), (332), $\bar{(2}11)$,(230), (343), (342), and (241) of NiFe_2_O_3_, respectively. These reflections are indexed to the rhombohedral structure of NiFe_2_O_3_ with the space group R3c, corresponding to hematite Fe_2_O_3_ (JCPDS card no. 01-073-2234) [23]. The absence of secondary or impurity phases indicates that no extraneous compounds were formed, confirming the successful incorporation of Ni into the Fe_2_O_3_ matrix without generating separate Ni or NiO phases. The XRD analysis also reveals that the introduction of dopant atoms, such as SiO_2_, RGO, and PIn, into the NiFe_2_O_3_ lattice modifies the crystalline structure without disturbing its fundamental rhombohedral phase. Notably, the $\bar{(1}10)$ peak shows a slight shift towards higher 2θ values upon doping, particularly for the RGO-NiFe_2_O_3_-SiO_2_-PIn sample, indicating a reduction in lattice spacing due to the substitutional doping effect. This peak shift can be attributed to changes in the lattice parameters due to the doping process, reflecting lattice contraction or expansion depending on the size and valence of the dopant ions. The systematic shift of diffraction peaks to higher angles suggests that introducing larger dopants (SiO_2_, RGO, and PIn) induces a reduction in the unit cell dimensions, implying lattice distortion. Moreover, the shift in the peak positions suggests that the crystal structure experiences internal stress or strain, which could combine compressive and tensile stresses depending on the nature of the doping interaction and the bonding environment. Furthermore, the broadening of XRD peaks, particularly for the (121) and $\bar{(1}10)$ reflections, reflects the nanoscale crystallite size of the materials. The average crystallite size for each nanocomposite was estimated using the Debye–Scherrer equation [24]. The calculated crystallite sizes for NiFe_2_O_3_, NiFe_2_O_3_-SiO_2_, RGO-NiFe_2_O_3_-SiO_2_, and RGO-NiFe_2_O_3_-SiO_2_-PIn are 13.82 nm, 14.15 nm, 16.62 nm, and 19.52 nm, respectively. This progressive increase in crystallite size with the introduction of SiO_2_, RGO, and PIn suggests an improvement in the material’s crystallinity. SiO_2_ and RGO may act as crystallization enhancers by promoting grain growth during the synthesis process, while PIn, being a polymer, could potentially facilitate the alignment of crystal domains. Additionally, the increasing crystallite size may reflect reduced structural disorder, as the inclusion of SiO_2_, RGO, and PIn could alleviate lattice defects or microstrains present in the pristine NiFe_2_O_3_ structure. The observed broadening of the (121) and $\bar{(1}10)$ peaks in the XRD patterns could also be attributed to microstrain or lattice distortion caused by the substitutional doping effect, where the larger dopant atoms (SiO_2_, RGO, and PIn) induce stress in the NiFe_2_O_3_ lattice. This lattice strain, in turn, could affect the material’s mechanical, magnetic, and catalytic properties by altering the local bonding environment and the distribution of electron density around the dopant sites. Furthermore, the absence of additional peaks or phase transformations indicates that the dopant atoms are well integrated into the NiFe_2_O_3_ nanoparticle, maintaining the rhombohedral hematite structure despite introducing new components. This suggests that the dopants are homogeneously distributed throughout the lattice rather than forming separate phases, which would have resulted in new diffraction peaks.

The FTIR spectra depicted in Figure 1C provide a detailed analysis of the structural and chemical changes within the nanocomposites of NiFe_2_O_3_, NiFe_2_O_3_-SiO_2_, RGO-NiFe_2_O_3_-SiO_2_, and RGO-NiFe_2_O_3_-SiO_2_-PIn across the wavenumber range of 4000 to 400 cm^−1^. Each spectrum reveals specific vibrational modes that correlate with the different components integrated into the nanocomposites. For the pristine NiFe_2_O_3_ (black curve), the absorption peaks around 580–600 cm^−1^ indicate the Fe–O bond vibrations fundamental to the spinel structure of NiFe_2_O_3_. These peaks signify the intact metal–oxygen framework in the material. Additional peaks at 1620 cm^−1^ and 3400 cm^−1^ are associated with O–H bending and stretching vibrations, respectively, pointing to the presence of adsorbed water or surface-bound hydroxyl groups [25]. The introduction of SiO_2_ in NiFe_2_O_3_-SiO_2_ (red curve) introduces new peaks at 1030–1100 cm^−1^, corresponding to Si–O–Si asymmetric stretching vibrations, indicating successful silica integration. However, the slight intensity variation in the Fe–O bond vibration suggests potential interactions between SiO_2_ and the NiFe_2_O_3_ surface, which might influence the local chemical environment and, subsequently, the properties of the composite. The sharper peak confirms the RGO incorporation in RGO-NiFe_2_O_3_-SiO_2_ (magenta curve) at 1630 cm^−1^, attributed to the C=C stretching vibrations of RGO [26]. The persistence of the Si–O–Si peak, alongside the appearance of a new weak band at 1380–1400 cm^−1^ for C–OH groups, points to an incomplete reduction in graphene oxide and a complex interaction of RGO with the other components, likely influencing the electrical properties of the composite. The addition of PIn in RGO-NiFe_2_O_3_-SiO_2_-PIn (blue curve) is distinctly marked by new bands around 1120 cm^−1^ and 1500–1600 cm^−1^, which are characteristic of C–N stretching and the C=C stretching vibrations from PIn’s quinoid and benzenoid rings, respectively [27]. These features suggest that PIn is well-integrated and likely contributes to enhanced electrical conductivity and electrochemical properties. The reduced intensity of the broad O–H stretching peak at 3400 cm^−1^ may indicate interactions between PIn and the hydroxyl groups, possibly affecting the composite’s surface reactivity and adsorption properties. Critically, while the fundamental structure of NiFe_2_O_3_ remains intact across the modifications, as evidenced by the consistent Fe–O stretching vibrations, SiO_2_, RGO, and PIn significantly alter the surface chemistry and introduce new functionalities. These changes are pivotal as they could potentially enhance the composite’s mechanical, thermal, and electrochemical properties. Moreover, the consistent observation of broad O–H stretching vibrations across all samples suggests that the surface chemistry is rich in hydroxyl groups or adsorbed water molecules, which could be advantageous or detrimental, depending on the intended application of the nanocomposites. Therefore, while the FTIR analysis confirms successful synthesis and integration of the various components, it also highlights the complexity of interactions within the composites that could influence their practical applications in adsorption and energy storage.

**Figure 2:**
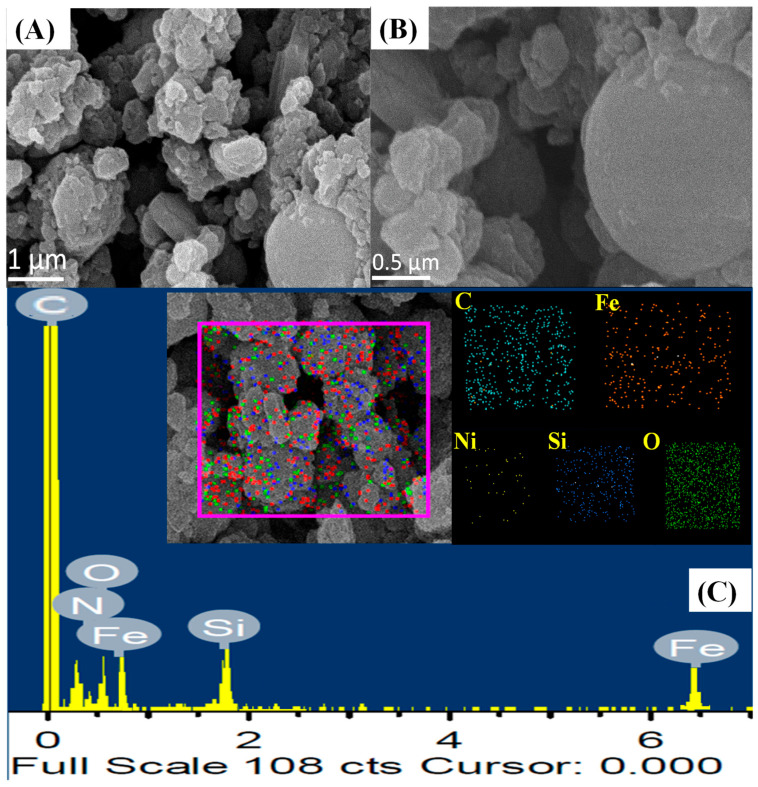
(**A**,**B**) SEM imaging, (**C**) elemental mapping.

The SEM images of the RGO-NiFe_2_O_3_-SiO_2_-PIn nanocomposite provide detailed insights into its structural and morphological characteristics. In Figure 2A, captured at a 1 μm scale, the composite exhibits a rough, porous surface with agglomerated nanoparticles. This porous morphology suggests a large surface area, enhancing its potential for adsorbing contaminants. The agglomeration of smaller particles forming larger clusters indicates a strong interaction between the composite’s various phases. In Figure 2B, at a 0.5 μm scale, a more detailed view of the nanoparticle arrangement is visible, where the closely packed nanoparticles form interconnected pores and channels. This intricate network facilitates the mass transfer and ion mobility, which is crucial for electrochemical performance in supercapacitor applications. Moreover, this porous network provides more available space for interactions, making the material suitable for adsorption processes. The EDX spectrum in Figure 2C further validates the composite’s elemental composition, with distinct peaks corresponding to C, O, Ni, Fe, Si, and N. Figure 2C inset represent the elemental mapping of the composite, demonstrating the homogeneous distribution of key elements such as carbon (C), nickel (Ni), iron (Fe), oxygen (O), and silicon (Si) across the material. This even distribution confirms the successful integration of reduced graphene oxide (RGO), NiFe_2_O_3_, SiO_2_, and polyindole (PIn) into a single cohesive composite.

**Figure 3:**
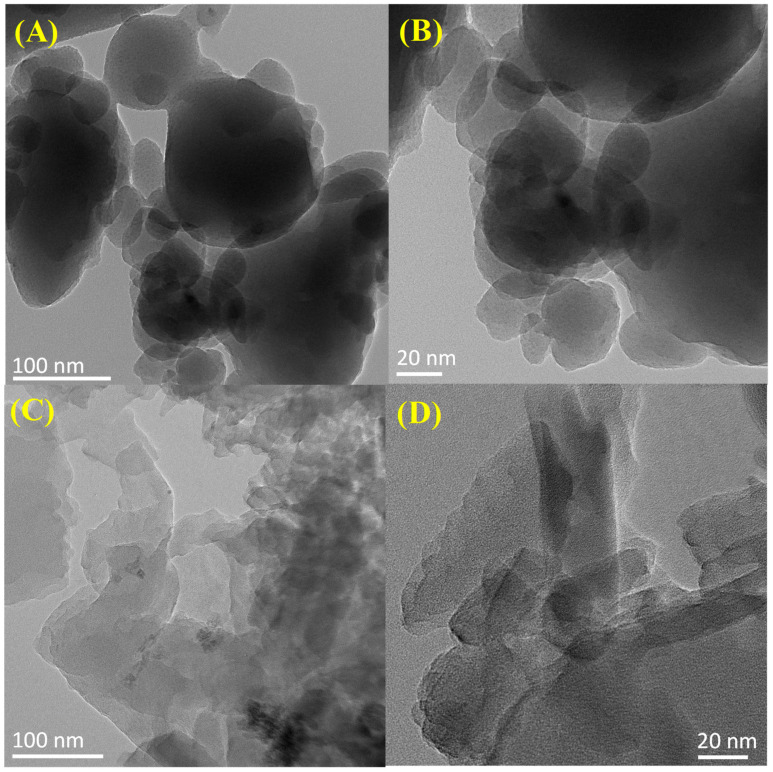
TEM imaging of (**A**,**B**) RGO-NiFe_2_O_3_-SiO_2_ and (**C**,**D**) RGO-NiFe_2_O_3_-SiO_2_-PIn.

The TEM images provide a detailed visualization of the structural characteristics of RGO-NiFe_2_O_3_-SiO_2_ and RGO-NiFe_2_O_3_-SiO_2_-PIn NCs at different scales. In Figure 3A, the TEM image of RGO-NiFe_2_O_3_-SiO_2_ at a 100 nm scale reveals well-aggregated NiFe_2_O_3_ nanoparticles dispersed uniformly across the RGO sheets. The close interaction between the nanoparticles and the RGO sheets suggests a strong binding between these components, likely enhancing the stability and conductive properties of the composite. Though SiO_2_ is not easily distinguishable at this scale, its presence likely provides added mechanical and thermal stability to the composite structure. Figure 3B, taken at a 20 nm scale, provides a clearer view of NiFe_2_O_3_ nanoparticles, with their distinct spherical morphology. At this higher resolution, the particles appear well dispersed across the RGO sheets, offering a high surface area beneficial for applications requiring efficient adsorption or electrochemical activity. The RGO sheets, visible as thin transparent layers, support the nanoparticles, reinforcing the strong interaction between the composite’s components. In Figure 3C,D, the TEM images of the RGO-NiFe_2_O_3_-SiO_2_-PIn NCs show how adding PIn affects the nanostructure. In Figure 3C, at a 100 nm scale, a more interconnected and intricate network is observed compared to the RGO-NiFe_2_O_3_-SiO_2_-PIn NCs. The polyindole likely enhances the polymeric framework, improving the composite’s flexibility while maintaining a porous structure. This interconnected network is advantageous for applications requiring efficient charge transport and ion diffusion, such as electrochemical devices. In Figure 3D, at a 20 nm scale, the interaction between NiFe_2_O_3_ nanoparticles and polyindole is more pronounced. The polyindole appears to form a thin coating around the nanoparticles, possibly contributing to improved electrochemical stability and charge transport. The nanoparticles remain well dispersed within the RGO matrix, with SiO_2_ continuing to provide structural reinforcement. The RGO sheets, visible as transparent layers, remain integral to supporting the composite structure and maintaining its conductive properties.

### 3.1. Adsorption Study

Figure S1 illustrates a progressive increase in Sb(III) removal efficiency across the tested materials: NiFe_2_O_3_-SiO_2_ (52.37%), RGO-NiFe_2_O_3_-SiO_2_ (85.37%), and RGO-NiFe_2_O_3_-SiO_2_-Pln NCs (91.84%). This increase in adsorption efficiency can be attributed to the structural and functional modifications introduced at each stage. The base material, NiFe_2_O_3_-SiO_2_, provides active sites for Sb(III) adsorption, but its capacity is limited by the available surface area and the lack of diverse functional groups to facilitate strong interactions with Sb(III). The introduction of reduced graphene oxide (RGO) significantly improves the performance by increasing the surface area and adding oxygen-containing functional groups, such as hydroxyl (-OH) and carboxyl (-COOH), which enhance Sb(III) binding through complexation, electrostatic interactions, and hydrogen bonding. Furthermore, RGO’s conductive properties promote better electron transfer, which strengthens the interaction between the adsorbent and Sb(III) ions. The addition of polyindole to the RGO-NiFe_2_O_3_-SiO_2_ composite further amplifies the adsorption efficiency. Polyindole introduces nitrogen-containing functional groups (-NH, -NH2), which provide additional binding sites for Sb(III) through complexation and chelation. These nitrogen groups, combined with the oxygen-containing groups from RGO, create a synergistic effect that maximizes the material’s adsorption capacity. Moreover, polyindole enhances the electron transfer properties of the nanocomposite, further improving the adsorption kinetics. Overall, the progressive increase in Sb(III) adsorption efficiency can be attributed to the combination of a larger surface area, the presence of additional functional groups, and enhanced electron transfer within the material, all of which contribute to stronger and more effective interactions with Sb(III) ions.

**Figure 4:**
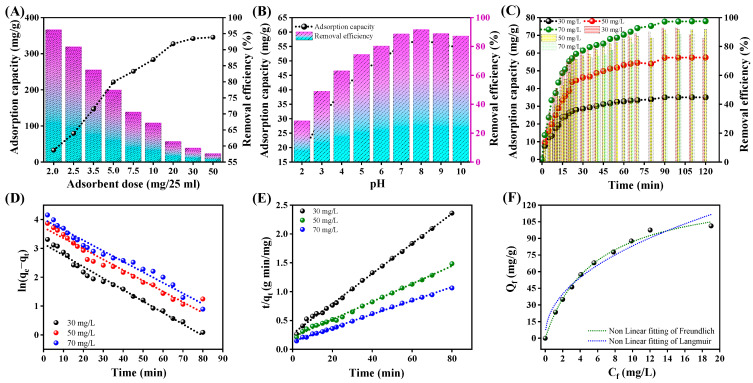
Effects on Sb(III) adsorption using RGO-NiFe_2_O_3_-SiO_2_-PIn NCs: (**A**) Variation with nanocomposite dose; (**B**) influence of pH; (**C**) impact of interaction time. Linear kinetics graphs: (**D**) pseudo-first order (**E**) pseudo-second order; (**F**) non-linear graph of Langmuir and Freundlich models.

The dosage of the adsorbent plays a critical role in determining its efficiency and significantly impacts the overall cost-effectiveness of the adsorption process. To assess the effect of RGO-NiFe_2_O_3_-SiO_2_-PIn NCs dosage on the adsorption capacity and removal efficiency of Sb(III) ions, varying amounts of adsorbent (ranging from 2 mg/25 mL to 50 mg/25 mL) were tested (Figure 4A). The results showed that as the adsorbent dosage increased, the adsorption capacity per unit mass decreased, while the overall removal efficiency improved. Specifically, when the RGO-NiFe_2_O_3_-SiO_2_-PIn NCs dosage was increased from 2 mg/25 mL to 20 mg/25 mL, adsorption capacity dropped from 366.83 mg/g to 57.40 mg/g, while removal efficiency increased from 58.69% to 91.84%. The reduction in adsorption capacity can be attributed to the decrease in concentration gradient between the adsorbent and adsorbate, leading to fewer metal ions being adsorbed per unit of adsorbent. Conversely, the increase in removal efficiency is likely due to the greater availability of functional groups and adsorption sites [28]. Beyond 20 mg/25 mL, further increases in adsorbent dosage did not significantly improve removal efficiency, suggesting that Sb(III) became the limiting factor in the system.

The pH of a solution is crucial for the effective removal of Sb(III) because it affects both the chemical form of the pollutant and the surface charge of the adsorbent. As shown in Figure 4B, the impact of pH on the adsorption of Sb(III) onto RGO-NiFe_2_O_3_-SiO_2_-PIn nanocomposites was studied under optimal conditions, with a contact time of 90 min, a fixed initial Sb(III) concentration of 50 mg/L, and an adsorbent dosage of 20 mg per 25 mL. Antimony can exist in three different forms based on pH: as the basic form Sb(OH)_4_^−^, the neutral Sb(OH)_3_, and the acidic Sb(OH)_2_^+^ [29]. The results showed that the removal efficiency of Sb(III) improved as the pH increased, reaching a peak at pH 8. This enhanced removal efficiency can be attributed to the speciation of Sb(III) in aqueous solution, where Sb(OH) _3_ becomes the dominant form at neutral to slightly alkaline pH values. Sb(OH)_3_, being neutral, does not experience electrostatic repulsion, which contrasts with negatively charged species like Sb(V), such as Sb(OH)_6_^−^, which may face repulsive forces when interacting with adsorbent surfaces. At pH 8, the neutral characteristic of Sb(OH)_3_ minimizes such repulsion, allowing it to interact more readily with the functional groups on the RGO-NiFe_2_O_3_-SiO_2_-PIn nanocomposites. As discussed in Zhou et al. [30], Sb(III) in the form of Sb(OH)_3_ is more stable and easier to remove in neutral and alkaline environments. This is primarily due to its neutral charge, which eliminates the electrostatic repulsion typically observed with charged species. In the Sb(V) removal processes, electrostatic repulsion between the negatively charged Sb(OH)_6_^−^ species and the similarly charged adsorbent surface can hinder the adsorption process. However, for Sb(III), the neutral Sb(OH)_3_ species can freely interact with surface-active sites on the nanocomposite, such as hydroxyl (-OH) and amino (-NH_2_) groups, without experiencing significant resistance. This leads to enhanced adsorption and removal efficiency at neutral to slightly alkaline pH levels [30]. Zhou et al. [30] also emphasize that while the theoretical basis for easier removal of Sb(III) due to its neutral form is strong, direct experimental evidence supporting this phenomenon has often been limited. The results observed in our study align with this hypothesis, providing additional experimental data that suggest the neutral form of Sb(III), Sb(OH)_3_, is indeed more effectively removed in these pH conditions. At lower pH levels, the protonation of surface groups such as –NH_2_ and –OH, along with the presence of excess H^+^ ions, creates electrostatic repulsion that hinders the adsorption of Sb(III). As the pH increases, the extent of protonation decreases, facilitating stronger interactions between Sb(III) and the adsorbent’s functional groups, enhancing the adsorption capacity. However, at pH levels exceeding 8, adsorption efficiency declines due to competition with hydroxide ions, which hampers Sb(III) adsorption and increases the likelihood of precipitated antimony species, reducing ion concentration in the solution and obstructing the adsorption process [31].

To determine the equilibrium time for adsorption, the removal efficiency of RGO-NiFe_2_O_3_-SiO_2_-PIn NCs for Sb(III) was evaluated over varying time intervals. As shown in Figure 4C, the adsorption rate of Sb(III) increased rapidly during the initial phase and gradually slowed as the process progressed, reaching equilibrium at around 90 min. This initial rapid adsorption was due to the abundant active sites on the surface of the adsorbent, allowing for easy capture of Sb(III) ions. As more ions were adsorbed, the number of available active sites decreased, leading to stronger repulsion between the adsorbed and free molecules, which slowed the rate of adsorption. Once all the adsorption sites were saturated, equilibrium was achieved. Figure 4C also shows that the equilibrium adsorption capacity of Sb(III) increases with increasing initial concentrations of metal ions. As noted by Chukwuemeka-okorie et al. [28], this increase in capacity is due to the stronger driving force between the solid and liquid phases as the concentration rises.

To elucidate the adsorption mechanism of Sb(III), the experimental data were analyzed using nonlinear pseudo-first-order and pseudo-second-order kinetic models. The corresponding fitting curves and key parameters are presented in Figure 4D,E and Table 1. The results indicated that the correlation coefficients (R^2^) for the pseudo-second-order model were consistently higher than those for the pseudo-first-order model. Additionally, the theoretical adsorption capacities (q_cal_) predicted by the pseudo-second-order model were much closer to the experimentally determined adsorption capacities (q_e_). This suggests that the adsorption process of Sb(III) is better described by the pseudo-second-order model, indicating that chemisorption plays a significant role in the adsorption process [32].

In this study, both the Langmuir and Freundlich models were applied to analyze the interactions between the pollutant and the adsorbent. The fitting curves and related parameters are shown in Figure 4F and Table 2. For Sb(III), the Langmuir model exhibited a higher R^2^ value compared to the Freundlich model, suggesting that the adsorption of Sb(III) is better described by the Langmuir model and follows a monolayer adsorption mechanism. At 303 K, the maximum adsorption capacity of RGO-NiFe_2_O_3_-SiO_2_-PIn NCs for Sb(III), based on the Langmuir model, was calculated to be 140.85 mg/g. Additionally, the separation factor ($R_{L}=\frac{1}{1+K_{L}C_{o}}$) was determined using the Langmuir model to further assess the feasibility of the adsorption process. The value of R_L_ indicates the adsorption behavior, where R_L_ > 1 is unfavorable; R_L_ = 1 is linear; 0 < R_L_ < 1 is favorable; and R_L_ = 0 is irreversible. In the adsorption of Sb(III) onto the RGO-NiFe_2_O_3_-SiO_2_-Pin NCs, R_L_ values were found to be less than 1 at all concentrations, indicating that the adsorption process was favorable and spontaneous. A comparison of the adsorption performance between RGO-NiFe_2_O_3_-SiO_2_-PIn NCs and other adsorbents is shown in Table 3, where it is evident that the RGO-NiFe_2_O_3_-SiO_2_-PIn NCs for the adsorption of Sb(III) presented comparable results to previously reported adsorbents [29,33,34,35,36,37,38,39]. Therefore, the synthesized RGO-NiFe_2_O_4_-SiO_2_-Pln NCs can be considered a highly effective adsorbent for the treatment of wastewater.

The influence of system temperature on the removal of Sb(III) using RGO-NiFe_2_O_3_-SiO_2_-PIn NCs was examined, with the corresponding fitting curves and data shown in Figure S2 and Table 4. As the temperature increased from 298 K to 323 K, the removal efficiency for Sb(III) improved from 84.75% to 97.43%, suggesting that higher temperatures enhanced the adsorption process. Additionally, the thermodynamic parameters, including the Gibbs free energy (ΔG), enthalpy change (ΔH), and entropy change (ΔS), are presented in Table 4. The results showed that the ΔG values were negative and decreased further, from −6.179 to −11.658 kJ/mol, as the temperature increased, confirming that the adsorption of Sb(III) occurred spontaneously [40]. The positive values of ΔH and ΔS indicated that the adsorption was endothermic and accompanied by an increase in entropy [41].

**Table 1:** Kinetic parameters for removal of Sb(III) onto RGO-NiFe_2_O_3_-SiO_2_-PIn NCs (Experimental parameters: RGO-NiFe_2_O_3_-SiO_2_-PIn NC dose = 20 mg/25 mL; temperature = 303 K; pH = 8).

Kinetics	Parameters	30 mg/L	50 mg/L	70 mg/L
P-fo	Q_exp_	34.995	57.40	77.719
	Q_fo_	23.575	41.434	55.329
	K_1_	0.0398	0.0369	0.0367
	R^2^	0.9802	0.9613	0.9728
P-so	Q_exp_	34.995	57.40	77.719
	Q_so_	38.610	65.359	86.207
	K_2_	2.437 × 10^−3^	1.097 × 10^−3^	0.962 × 10^−3^
	R^2^	0.9981	0.9966	0.9979

**Table 2:** Isotherm model factors for the elimination of Sb(III) onto RGO-NiFe_2_O_3_-SiO_2_-PIn NCs (Experimental parameters: RGO-NiFe_2_O_4_-SiO_2_-Pln NC dose = 20 mg/25 mL; temperature = 303 K; pH = 8).

Isotherms	Parameters	Sb(III)
Langmuir	Q_L_	135.898
	K_L_	0.1777
	R^2^	0.9952
Freundlich	K_F_	29.017
	n_F_	0.4573
	R^2^	0.94301

**Table 3:** Comparison with the reported literature data for the adsorption of Sb(III) onto the various adsorbents.

Adsorbents	Adsorption Capacity (mg/g)	PH	Dose (g/L)	Ref.
Mercapto-functionalized, silica-supported organic–inorganic hybrid sorbent	108.8	5	5	[33]
Thiol-functionalized MnFe_2_O_4_-MCM-41	164.8	7	1.5	[29]
RGO-Au-Ag_2_O/PIn NCs	121.62	8	0.6	[34]
rGO aerogel	168.59	6	1	[35]
Polyamide–graphene	158.2	7	1.5	[36]
RGO/Mn_3_O_4_	120.3	6.8	1	[37]
Nano-modified chitosan (Nano-Fe_3_O_4_, carboxylate metal organic frameworks (MOFs))	69.85	11	1.5	[38]
Co_3_O_4_@rGO	151.1	7	1	[39]
α-MnO_2_ nanofibers	111.70	6	0.5	[42]
Hematite-modified magnetic nanoparticles	36.7	4.1	0.1	[43]
RGO-NiFe_2_O_3_-SiO_2_-PIn	135.898	8	0.8	Our work

**Table 4:** Thermodynamic factors for the removal of Sb(III) onto RGO-NiFe_2_O_3_-SiO_2_-PIn NCs (Experimental parameters: RGO-NiFe_2_O_3_-SiO_2_-PIn NC dose = 20 mg/25 mL; pH = 8; concentration of Sb(III) = 50 mg/L).

T (K)	∆S (J/K/mol)	∆H (kJ/mol)	∆G (kJ/mol)	R^2^
298	215.324	57.621	−6.179	0.9679
303			−7.953	
313			−10.082	
323			−11.658	

**Scheme 1:**
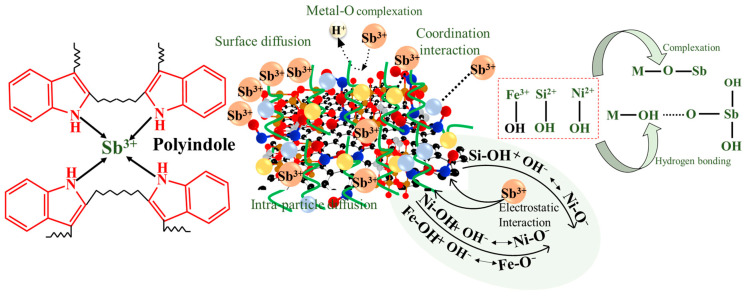
The adsorption mechanism of Sb(III) onto the RGO-NiFe_2_O_3_-SiO_2_-PIn NCs.

The surface of RGO-NiFe_2_O_3_-SiO_2_-PIn NCs may possess active sites or functional groups capable of attracting and binding Sb(III) ions via various chemical bonding mechanisms, including electrostatic attraction, ion exchange, or complexation, as illustrated in Scheme 1 [44]. The FTIR spectrum of RGO-NiFe_2_O_3_-SiO_2_-Pln NCs after adsorption of Sb(III) (Figure S3) reveals several shifts and new features, indicating the interaction between the nanocomposite and Sb species. The O–H stretching peak around 3400 cm^−1^ shows broadening, suggesting hydrogen bonding or interaction between surface hydroxyl groups and the adsorbed Sb species. The C=C stretching peak near 1500–1600 cm^−1^ exhibits a slight shift, which points to possible interaction or complexation between the sp^2^ carbon network of RGO and Sb(III) ions. The characteristic Si–O–Si peak around 1380–1400 cm^−1^ and Fe–O vibration at 580–600 cm^−1^ shows a slight change in the intensity of peaks, suggesting their involvement in the adsorption process. Notably, new weak bands appear around 600–700 cm^−1^, potentially corresponding to Sb–O bonds, which are indicative of antimony being successfully adsorbed onto the composite material [45]. These spectral changes provide clear evidence of the successful adsorption of Sb species onto the RGO-NiFe_2_O_3_-SiO_2_-Pln NCs, primarily through surface interactions and potential chemical bonding with the functional groups of the composite.

**Figure 5:**
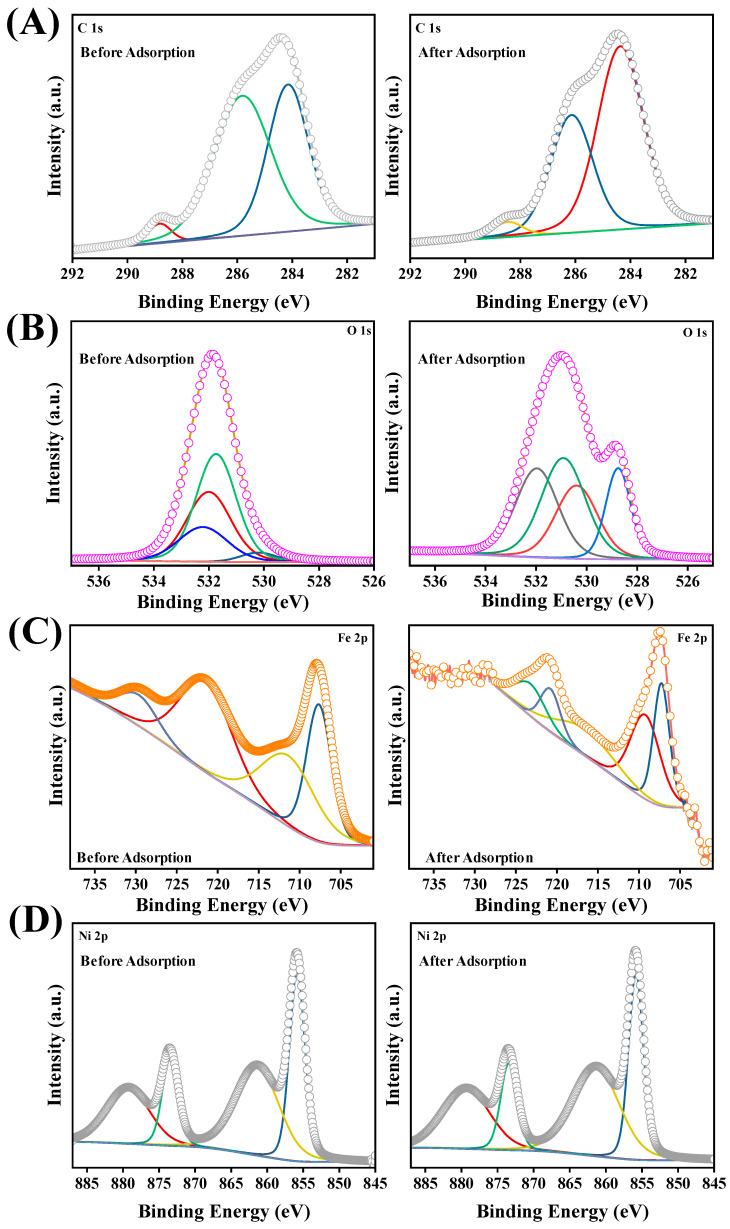

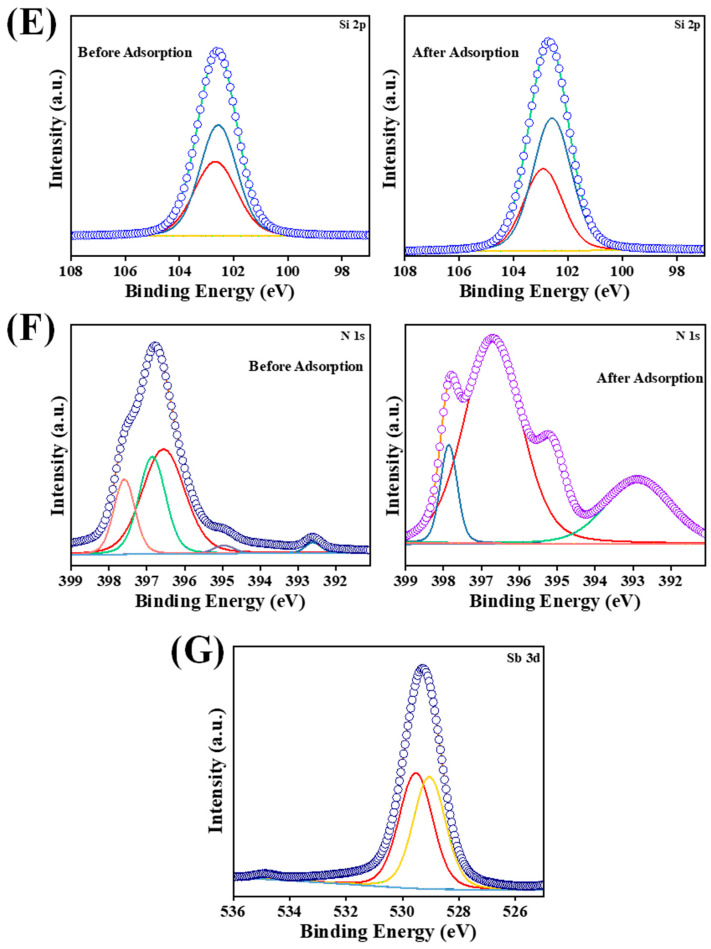
XPS of RGO-NiFe_2_O_3_-SiO_2_-PIn NCs before and after adsorption of Sb(III): (**A**) C 1s, (**B**) O 1s, (**C**) Fe 2p, (**D**) Ni 2p, (**E**) Si 2p, (**F**) N 1s, (**G**) Sb 3d.

The XPS analysis provides detailed insights into the surface chemistry of the RGO-NiFe_2_O_3_-SiO_2_-PIn NCs before and after the adsorption of Sb(III). For C 1s (Figure 5A), the main peak observed at 284.8 eV before adsorption corresponds to C–C/C=C bonds, indicative of the sp^2^ hybridized carbon framework in RGO [46]. Additional peaks at 286.4 eV and 288.6 eV represent C–O and C=O bonds, respectively [47]. These oxygenated functional groups suggest residual oxygen species in the RGO structure. After Sb(III) adsorption, these peaks exhibit slight intensity changes, indicating interaction of the carbon surface with adsorbed Sb(III), possibly through physical adsorption or minor electronic rearrangements (Figure 5A). For O1s (Figure 5B), the dominant peak around 531.5 eV before adsorption corresponds to metal–oxygen bonds, such as in NiFe_2_O_3_ or SiO_2_, with a smaller peak at 533.0 eV associated with hydroxyl groups or C-O bonds in RGO [48]. After adsorption, the increase in intensity of the peak at 531.5 eV suggests a higher density of oxygen–metal bonds, likely due to the interaction of oxygen with Sb(III) species during adsorption (Figure 5B). This points to oxygen atoms in the composite playing a crucial role in Sb binding, possibly through surface coordination or complex formation. The Fe 2p spectrum (Figure 5C) shows Fe 2p3/2 and Fe 2p1/2 peaks at 709.5 eV and 724.0 eV, respectively, along with satellite peaks, indicating Fe^3+^ in the composite, which likely arises from NiFe_2_O_3_ [49]. After adsorption, a slight shift in the Fe 2p peaks and changes in satellite intensity suggest interaction between Fe sites and Sb species, potentially through complexation or redox activity. This indicates that Fe plays a role in the adsorption process, possibly through electron transfer or surface complexation (Figure 5C). The Ni 2p peaks at 855.5 eV (Ni 2p3/2) and 873.0 eV (Ni 2p1/2), along with satellite peaks, are characteristic of Ni^2+^ in the composite [50], likely from NiFe_2_O_3_ (Figure 5D). After Sb(III) adsorption (Figure 5D), these peaks undergo subtle changes in intensity and position, implying the involvement of nickel in the adsorption process. The binding energy changes could indicate electronic interaction between nickel and the adsorbed Sb(III) species, suggesting nickel may facilitate adsorption through coordination or redox mechanisms. The Si 2p peak at 103.5 eV corresponds to Si^4+^ in SiO_2_ [51], and this remains largely unchanged after Sb(III) adsorption (Figure 5E). This stability suggests that silicon is not directly involved in the adsorption process and remains inert during Sb(III) binding. The role of SiO_2_ is likely structural, contributing to the composite’s stability without actively participating in Sb adsorption. In the N 1s spectrum (Figure 5F), a peak at 397.6 eV before adsorption is assigned to nitrogen in polyindole, particularly amine or imine groups (C-N or C=N bonds) [50]. After adsorption, significant changes in the intensity and shape of this peak suggest that nitrogen groups in polyindole interact with Sb(III), likely through coordination bonding. The nitrogen functionality in polyindole appears to be an active site for Sb(III) adsorption, which is critical for the material’s adsorption efficiency. Finally, the Sb 3d spectrum (Figure 5G) observed after adsorption shows Sb 3d5/2 at approximately 529.0 eV, which is consistent with Sb(III) [52]. The binding energy confirms that Sb(III) has been successfully adsorbed onto the nanocomposite surface. The clear appearance of Sb peaks after adsorption provides strong evidence of the material’s efficacy in capturing Sb(III), likely through surface complexation or adsorption onto functional groups present on the composite. In summary, the XPS spectra indicate that adsorption of Sb(III) onto the RGO-NiFe_2_O_3_-SiO_2_-PIn NCs involves multiple components, including Fe, Ni, oxygen, and nitrogen (Scheme 1). The changes in binding energy and peak intensity for these elements after adsorption suggest that surface interactions, particularly with oxygen and nitrogen functionalities, as well as potential redox reactions involving Fe and Ni, play critical roles in the efficient binding of Sb(III). This multi-site adsorption mechanism likely enhances the overall performance of the composite, making it a promising material for Sb(III) removal from contaminated water.

### 3.2. Electrochemical Supercapacitor Analysis of Spent RGO-NiFe_2_O_3_-SiO_2_-PIn NCs

**Figure 6:**
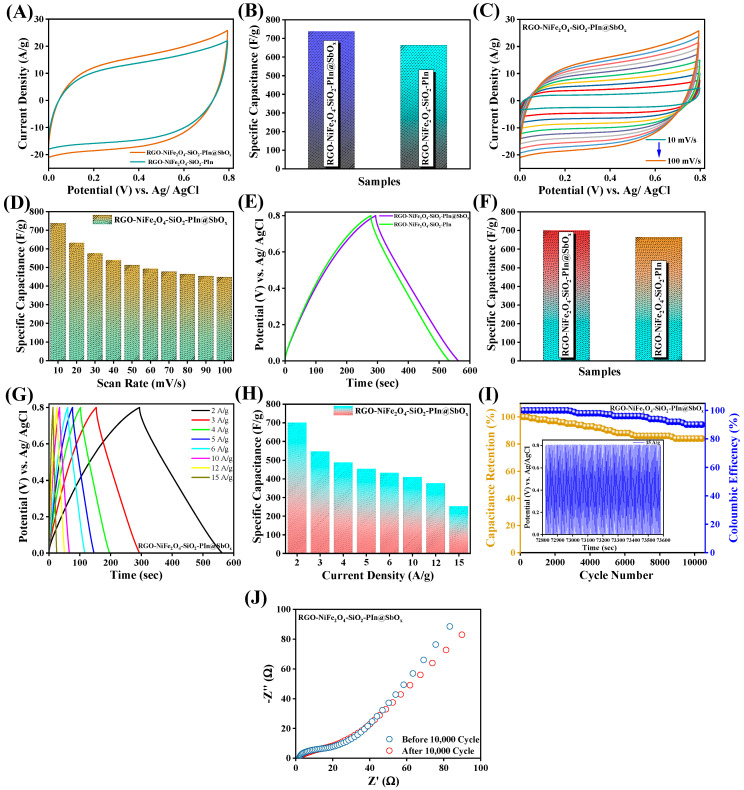
(**A**,**B**) Comparative CV curve and C_s_ of RGO-NiFe_2_O_3_-SiO_2_-PIn and RGO-NiFe_2_O_3_-SiO_2_-PIn@SbO_x_ at 100 mV/s. (**C**) CV curve of RGO-NiFe_2_O_3_-SiO_2_-PIn@SbO_x_ at various scanning speeds. (**D**) C_s_ of RGO-NiFe_2_O_3_-SiO_2_-PIn@SbO_x_ at various scanning speeds (10 to 100 mV/s). (**E**,**F**) Comparative GCD curve and C_s_ of RGO-NiFe_2_O_3_-SiO_2_-PIn and RGO-NiFe_2_O_3_-SiO_2_-PIn@SbO_x_ at 2 A/g. (**G**) GCD curve of RGO-NiFe_2_O_3_-SiO_2_-PIn and RGO-NiFe_2_O_3_-SiO_2_-PIn@SbO_x_ at various current densities, (**H**) C_s_ of RGO-NiFe_2_O_3_-SiO_2_-PIn@SbO_x_ at various current densities (2 to 15 A/g). (**I**) Capacitive retention values of RGO-NiFe_2_O_3_-SiO_2_-PIn@SbO_x_ at 15 A/g for 10,000 cycles. (**J**) Nyquist plots of RGO-NiFe_2_O_3_-SiO_2_-PIn@SbO_x_.

The utilization of Sb(III)-adsorbed RGO-NiFe_2_O_3_-SiO_2_-PIn in supercapacitor applications was investigated in order to reduce the risk of additional pollution arising from the direct disposal of adsorbents saturated with pollutants. Antimony is known for its high stability and admirable carrier mobility, efficient charge transport, durability, and favorable electrical resistivity. Using Sb-adsorbed RGO-NiFe_2_O_3_-SiO_2_-PIn in supercapacitor applications significantly enhances the value of spent adsorbents [53]. This research thoroughly assessed the electrochemical properties of RGO-NiFe_2_O_3_-SiO_2_-PIn and RGO-NiFe_2_O_3_-SiO_2_-PIn@SbOx electrodes within a three-electrode setup, utilizing a 1 M Na_2_SO_4_ electrolyte. The CV curves for RGO-NiFe_2_O_3_-SiO_2_-PIn and RGO-NiFe_2_O_3_-SiO_2_-PIn @SbO_x_ are depicted in Figure 6A. The curves were scanned at a rate of 100 mV/s within a potential range of 0 to 0.8 V. The CV examination suggests a hybrid capacitive behavior, which is likely the consequence of a combination of pseudocapacitance and electric double-layer capacitance (EDLC) characteristics [34]. Moreover, the area covered by the CV curve of RGO-NiFe_2_O_3_-SiO_2_-PIn@SbO_x_ is much more compared to RGO-NiFe_2_O_3_-SiO_2_-PIn. As the C_s_ is the function of area covered by the CV curve and directly proportional to C_s_, the RGO-NiFe_2_O_3_-SiO_2_-PIn@SbO_x_ electrode material comprises highest C_s_ of 738.27 F/g in comparison to the RGO-NiFe_2_O_3_-SiO_2_-PIn (664.45 F/g).

The CV curves for the RGO-NiFe_2_O_3_-SiO_2_-PIn@SbO_x_ at variations in scan rate from 10 to 100 mV/s depicted in Figure 6C show the growth of the area linked to the CV curves along with the intensification of the scan rates. It supports the improved current capability along with the rate performance of the electrode. The peak current varies directly with the increase in the scan rate, which indicates that the storage mechanism of energy at the electrode–electrolyte edge has good reversibility. The specific capacity (Cs) for RGO-NiFe_2_O_3_-SiO_2_-PIn@SbO_x_ and the respective values at different scan rates of 10, 20, 30, 40, 50, 60, 70, 80, 90, and 100 mV/s were about 738.27, 632.37, 576.41, 539.86, 513.64, 493.67, 478.29, 465.41, 454.56, and 449.05 F/g, respectively (Figure 6D). Figure 6D shows that the C_s_ of the RGO-NiFe_2_O_3_-SiO_2_-PIn@SbO_x_ decreases with the increasing scan rate. The lower capacitance can be explained by the existence of inner active sites, which are unable to completely maintain redox transitions at higher scan rates [54]. The remarkable C_s_ of RGO-NiFe_2_O_3_-SiO_2_-PIn@SbO_x_ NCs could be credited to the synergistic effects of RGO, NiFe_2_O_3_-SiO_2_, and Pln, as well as due to the presence absorbed antimony on the surface of RGO-NiFe_2_O_3_-SiO_2_-PIn NCs. Antimony added to the surface of the RGO-NiFe_2_O_3_-SiO_2_-Pln may increase its capacitance properties, resulting in improved energy storage.

Figure 6E shows the GCD curves of RGO-NiFe_2_O_3_-SiO_2_-PIn and RGO-NiFe_2_O_3_-SiO_2_-PIn@SbO_x_ at a current density of 2 A/g. The symmetric nature between the charging part and their corresponding discharge part in the GCD analysis indicates the excellent reversibility as well as good electrochemical capacitive behavior of the composites. The RGO-NiFe_2_O_3_-SiO_2_-PIn@SbO_x_ NCs demonstrate extended discharge durations compared to RGO-NiFe_2_O_3_-SiO_2_-PIn, suggesting that antimony adsorption augments the electrochemical characteristics of RGO- RGO-NiFe_2_O_3_-SiO_2_-PIn NCs. The supercapacitor performances of the composites were further investigated by determining the C_s_. Unsurprisingly, the RGO-NiFe_2_O_3_-SiO_2_-PIn@SbO_x_ (701.36 F/g) shows the best C_s_ value as compared to RGO-NiFe_2_O_3_-SiO_2_-PIn 664.45 F/g at 2 A/g. Figure 6F depicts the GCD curves of the RGO-NiFe_2_O_3_-SiO_2_-PIn@SbO_x_ NCs at varying current densities ranging from 2 to 15 A/g. The capacitance values that correspond to the specified current densities are shown in Figure 6G. It was determined that these values were 701.36, 547.59, 487.96, 454.38, 431.83, 410.21, 375.92, and 253.96 F/g at 2, 3, 4, 5, 6, 10, 12, and 15 A/g, respectively from the charge–discharge profile. The losses in C_s_ at high current densities indicate a reduction in electrochemical activity and performance because the electrolyte ions are unable to reach electroactive sites and regions. GCD measurements at 15 A/g for 10,000 cycles for RGO-NiFe_2_O_3_-SiO_2_-PIn@SbO_x_ electrode materials have been carried out, and the obtained capacity retention as a function of the cycle number is demonstrated in Figure 6G. From this figure, it is clearly seen that capacity retention of the RGO-NiFe_2_O_3_-SiO_2_-PIn@SbO_x_ electrode materials after 10,000 cycles retained up to 80.15% capacity. This capacity retention of the composite material verified chemical and structural optimization within the electrode material for long-term sustainability of electrochemical reactions. The EIS technique was used to study the resistance and capacitive nature, reaction at electrode–electrolyte interface, etc., of the prepared electrode over the frequency range of 0.01 Hz to 100 kHz. Figure 6H presents the Nyquist graph of the RGO-NiFe_2_O_3_-SiO_2_-PIn@SbO_x_. The solution resistance (R_s_) value was determined to be 5.46 Ω while the charge transfer resistance (R_ct_) value was calculated to be 8.25 Ω. The values of R_s_ and R_ct_ are small, which supports the idea of the easy and fast movement and diffusion of ions inside the porous structure of the synthesized nanocomposites. The outcomes point out that the synergistic effect of adsorbed antimony, RGO, NiFe_2_O_3_-SiO_2_, and PIn in the RGO-NiFe_2_O_3_-SiO_2_-PIn@SbO_x_ improves its electrical conductivity, resulting in an improvement in its electrochemical performance.

## 4. Conclusions

In summary, our study introduces a novel strategy to address two critical issues in environmental sustainability and energy storage. By utilizing RGO-NiFe_2_O_3_-SiO_2_-PIn, we have developed a multifunctional approach that not only efficiently removes Sb(III) contaminants from industrial wastewater but also repurposes the used adsorbent for supercapacitor energy storage. The synthesized RGO-NiFe_2_O_3_-SiO_2_-PIn exhibited excellent adsorption properties, achieving a removal efficiency of around 91.84% at an initial Sb(III) concentration of 50 mg/L and pH 8. The adsorption behavior followed the pseudo-second-order kinetic model and the Langmuir adsorption isotherm, confirming the reliability of the process. Moreover, the Sb-laden adsorbent (RGO-NiFe_2_O_3_-SiO_2_-PIn@SbO_x_) was successfully applied in energy storage, delivering a C_s_ of 701.36 F/g at a current density of 2 A/g. The material maintained 80.15% of its capacitance after 10,000 charge–discharge cycles, demonstrating its long-term stability and robustness. Overall, this research presents an innovative approach by integrating waste treatment with waste utilization, showing the potential to recycle antimony-containing waste adsorbents for energy storage. This dual-functionality solution not only tackles industrial wastewater pollution but also contributes to sustainable energy storage, representing a significant advancement toward a greener future.

## Data Availability

Data are contained within the article and Supplementary Materials.

## Supplementary Materials

The following supporting information can be downloaded at: https://www.mdpi.com/article/10.3390/polym16213084/s1, Figure S1: Removal efficiency of Sb(III) onto the RGO-NiFe_2_O_3_-SiO_2_-Pln, RGO-NiFe_2_O_3_-SiO_2_ and NiFe_2_O_3_-SiO_2_; Figure S2: lnKc vs 1/T plot for the adsorption of Sb(III) onto the RGO-NiFe_2_O_3_-SiO_2_-PIn NCs; Figure S3: FTIR of RGO-NiFe_2_O_3_-SiO_2_-PIn NCs before and after adsorption of Sb(III). References [55,56] are cited in the Supplementary Materials.
