# Supplementary material for: Polyindole-Functionalized RGO-NiFe2O4-SiO2 Nanocomposite: A Dual-Functional Nanomaterial for Efficient Antimony Adsorption and Subsequent Application in Supercapacitor
Source: Polymers (Basel). 2024 Oct 31;16(21):3084. doi: 10.3390/polym16213084 (PMC11548570; doi:10.3390/polym16213084)
Supplement: Supplementary file 1 [file polymers-16-03084-s001.zip › polymers-3254245-supplementary.pdf]

Supplementary Material

Polyindole-Functionalized RGO-$NiFe_2O_4$-SiO_2 Nanocomposite: A Dual-Functional Nanomaterial for Efficient Antimony Adsorption and subsequent application in Supercapacitor

Mohd Shoeb[#], Fouzia Mashkoor[#], Mohmmad Naved Khan, Changyoon Jeong

School of Mechanical Engineering, Yeungnam University, Gyeongsan, Gyeongbuk, 38541 Republic of Korea

[#]Authors have equal contribution in this manuscript.

Corresponding Author

Dr. Changyoon Jeong
Email: yoonni22@yu.ac.kr

School of Mechanical Engineering,
Yeungnam University,
Gyeongsan, Gyeongbuk, 38541
Republic of Korea

1S. Materials

All the chemical reagents used in this research work were of analytical reagent (AR) grade from from Sigma Aldrich, South Korea.

2S. Instrumental Characterization

The crystallinity phase and unit cell dimension were determined using a Shimadzu LabX XRD 6100 advance diffractometer (Anode material Cu, current of 30 mA, the voltage of 40 kV, wavelength (Cu) 1.541838). Optics: Beta filtering with graphite, automatic divergence slit, and monochromator for RGO-$NiFe_2O_3$-SiO_2-PIn NCs in the angle 2θ the range varies from 20.0° to 70.0° in 0.04° steps for RGO- $NiFe_2O_3$-SiO_2-PIn NCs . Surface morphology and element identification were investigated using a scanning electron microscope (Model No. JSM 6510 LV, Make-JEOL, Japan) equipped with an energy dispersive X-ray spectrometer (EDAX). Transmission electron microscopy (TEM) and selected area electron diffraction (SAED) patterns were used to study nanoparticles' particle size, shape, crystalline structure, and surface morphology. Before precipitating on the grid, powder sample of RGO- $NiFe_2O_3$-SiO_2-PIn NCs were suspended in ethanol. The sample was then directed before being scanned by TEM (Model No. JEM 2100,- JEOL, Japan). For determining the particle size distribution software version (1.4.3.6.7) made in Tokyo, Japan, broken-symmetry, Image J Launcher was used. For analyzing the functional molecules, RGO- $NiFe_2O_3$-SiO_2-PIn NCs were recorded using Fourier transform infrared (FTIR) spectroscopy at wavenumbers ranging from 400 to 4000 cm-1 (Model: Tensor 35, Make: Bruker) was used to examine the infrared spectra of NCs. Each sample was made by combining 200 mg of KBr (which served as a non-absorbing medium in the background) and compressing it under high pressure. Inductively Coupled Plasma-Atomic Emission Spectroscopy,

ICP-AES (OPTIMA 8300, Perkin Elmer) was used to analysis the concentration of heavy metal ion in a solution.

3S. Batch adsorption studies

The RGO-$NiFe_2O_3$-SiO_2-PIn NCs as an adsorbents for Sb(III) ion removal experiments were performed as follow. Typically, 20.0 mg of the RGO-$NiFe_2O_3$-SiO_2-PIn NCs were mixed with 25.0 mL of 50 mg/L concentration of Sb(III) solution at room temperature. The initial pH value of Sb(III) solution was adjusted to 8. At pre-set intervals, a certain amount of the suspension was withdrawn from the system. The supernatant obtained from different intervals was monitored by using Inductively Coupled Plasma-Atomic Emission Spectroscopy, ICP-AES (OPTIMA 8300, Perkin Elmer) to confirm the residual Sb(III) in the system. The adsorbed amount (q_t/q_e) onto the RGO-$NiFe_2O_3$-SiO_2-PIn NCs was determined from the change in the concentrations of Sb(III) at any time/equilibrium (t/e) by using following equation (Equation S1):

$$q_{t/e} = \left(C_o - C_{t/e}\right) \times \frac{V}{m} \tag{S1}$$

The removal efficiency (%R) was obtained by using the following expression (Equation S2):

$$\%R = \frac{\left(C_0 - C_{t/e}\right)}{C_0} \times 100 \tag{S2}$$

where, C_o/C_t/C_e (mg/L) signifies the initial/any time/equilibrium liquid-phase concentrations of solute, respectively.

The pseudo-first order, and pseudo-second order models are described in Equations S3-S4 [1,2]. In these equations, k_1, and k_2 are the equilibrium rate constants of the pseudo-first order, and pseudo-

second order models, respectively, while Q_{fo} and Q_{so} represent the equilibrium adsorption capacities (mg/g) of the pseudo-first order, and pseudo-second order models, respectively.

Linear P-fo: $$\ln(Q_f - Q_t) = lnQ_{f_o} - K_1 t \quad \text{(S3)}$$

Linear P-so: $$\frac{t}{Q_t} = \frac{1}{K_2 Q_{so}^2} + \frac{t}{Q_{so}} \quad \text{(S4)}$$

The experimental data was fitted using two adsorption isotherms models namely, Langmuir, and Freundlich models to theoretically explore the adsorption process. Nonlinear form of these isotherm models are expressed in Equations S5-S6. All adsorption isotherm models were used by the nonlinear form of Q_f versus C_f. Here, Q_L, is a maximum adsorption capacity (mg/g) of Langmuir. Meanwhile, K_L (L/mol), and K_F (L/mg) respectively, are the constants of Langmuir, and Freundlich models, which are related to the adsorption capacity or the interaction between the adsorbent and the adsorbate. Besides, n_F is a constant of Freundlich expressions, which represent the adsorption intensity. R (8.314 J mol/K), and T, are the universal gas constant, and temperature in Kelvin, respectively.

$$Q_f = \frac{Q_L K_L C_f}{1 + K_L C_f} \quad \text{S5}$$

$$Q_f = K_F C_f^{\frac{1}{n_F}} \quad \text{S6}$$

In these isotherms, the Langmuir model (Equation S5) and the empirical Freundlich formula (Equation S6) are the monolayer sorption on the uniform surface and multilayer sorption on the heterogeneous surface, respectively.

The thermodynamic parameters i.e., Gibb's free energy (ΔG), enthalpy (ΔH), and entropy (ΔS) were computed by the Equation S7 and S8

$$\Delta G = -RT\ln K_c \quad \text{(S7)}$$

$$\ln K_c = \frac{-\Delta G}{RT} = \frac{\Delta S}{R} - \frac{\Delta H}{RT} \quad \text{(S8)}$$

where, K_c is the thermodynamic constant and can be estimated by the ratio of adsorption capacity to the residual concentration of metal ion molecule in the solution.

4S. Electrochemical studies

Firstly, the spent-adsorbent comprising Sb(III) were further calcined at 300 °C for 1 h. The as-synthesized sample was named RGO-$NiFe_2O_3$-SiO_2-PIn NCs@SbO_x. The Corrtest CS350 electrochemical workstation (Ccs Studio 5; version 5.5.618.2) was used to conduct the electrochemical tests for three electrode measurements we performed on an electrochemical workstation. The current experiment utilizes three electrodes. The platinum wire acts as a counter electrode, Ag/AgCl is a reference electrode, and carbon cloth (1 cm x 2 cm) is the working electrode. All electrochemical experiments were performed with sodium sulfate (1 M Na_2SO_4) as the electrolyte. The electrode material's specific capacitance was calculated based on CV voltammograms and galvanostatic charge-discharge curves. Using data from galvanostatic charge/discharge curves, the following Equation S9, and S10 were used to determine the specific capacitance of the electrode material.

$$C = \frac{\int I\, dv}{mv\Delta V} \quad \text{S9}$$

$$C_{sp} = \frac{I\Delta t}{\Delta V \times m} \quad \text{S10}$$

Where C signifies the specific capacitance from CV, C_{sp} signifies the specific capacitance from GCD, ΔV (voltage) is the restricted voltage window, I (A) represents the current acquired from the cyclic voltammetry curve, Δt is the time duration of one complete cycle, ν is the time-rate of change of voltage or scan rate, m is the mass of the sample deposited on the surface of electrode.

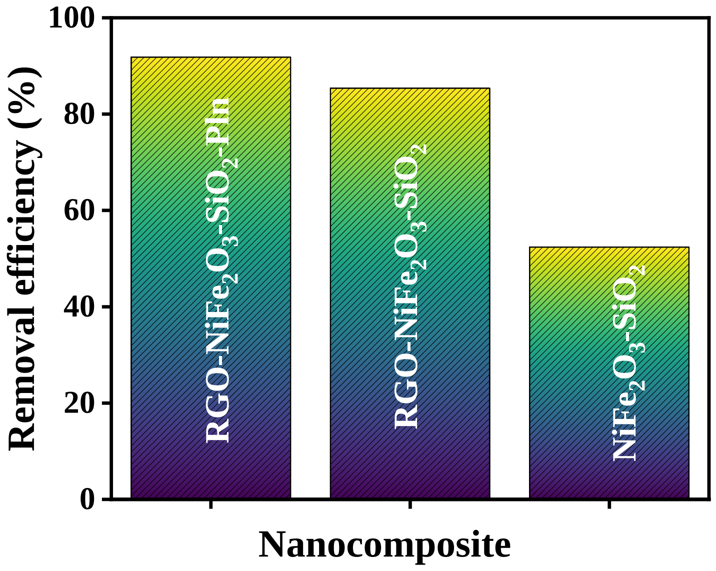

Figure S1. Removal efficiency of Sb(III) onto the RGO-$NiFe_2O_3$-SiO_2-Pln, RGO-$NiFe_2O_3$-SiO_2 and $NiFe_2O_3$-SiO_2

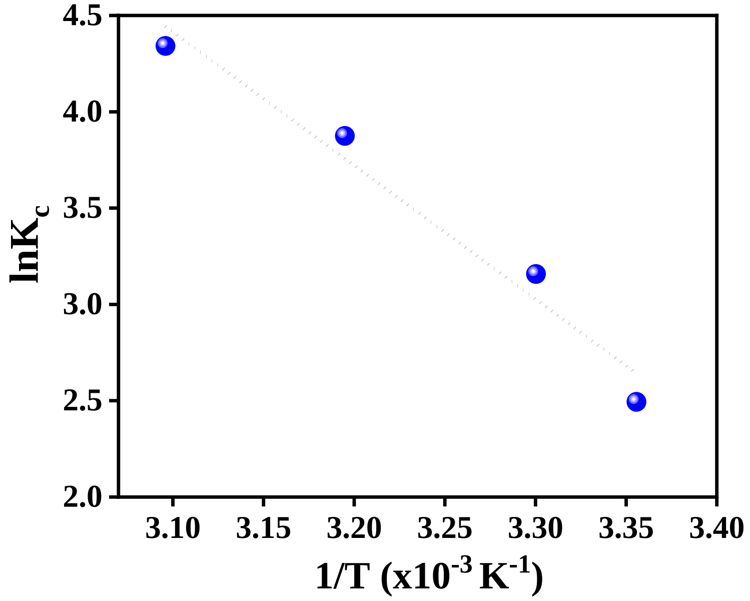

Figure S2. lnK_c vs 1/T plot for the adsorption of Sb(III) onto the RGO-$NiFe_2O_3$-SiO_2-PIn NCs.

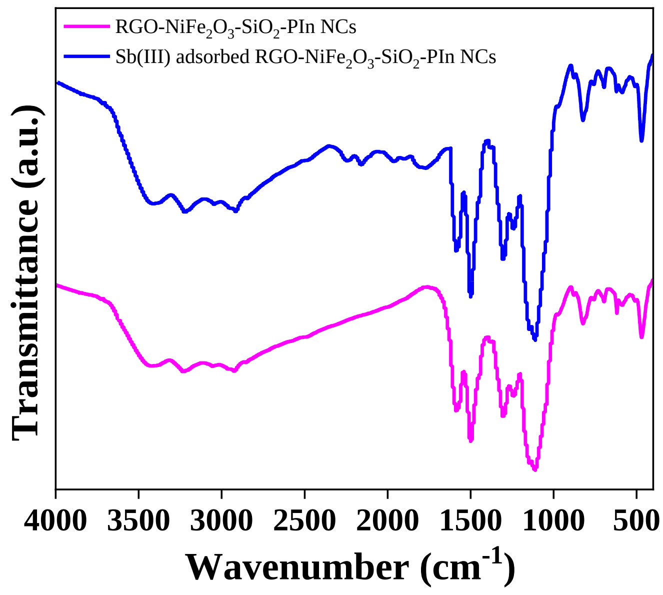

Figure S3. FTIR of RGO-$NiFe_2O_3$-SiO_2-PIn NCs before and after adsorption of Sb(III).

References

1. Xiong, C.; Wang, S.; Zhang, L. Selective recovery mechanism of Au(III) from an aqueous solution by trimethyl phosphate modified poly(glycidyl methacrylate). *Journal of the Taiwan Institute of Chemical Engineers* **2019**, *95*, 55-64, doi:https://doi.org/10.1016/j.jtice.2018.09.035.

2. Xiong, C.; Wang, S.; Hu, P.; Huang, L.; Xue, C.; Yang, Z.; Zhou, X.; Wang, Y.; Ji, H. Efficient selective removal of Pb (II) by using 6-aminothiouracil-modified Zr-based organic frameworks: from experiments to mechanisms. *ACS applied materials & interfaces* **2020**, *12*, 7162-7178.
